# Patient- and therapy-related factors associated with the incidence of xerostomia in nasopharyngeal carcinoma patients receiving parotid-sparing helical tomotherapy

**DOI:** 10.1038/srep13165

**Published:** 2015-08-20

**Authors:** Tsair-Fwu Lee, Ming-Hsiang Liou, Hui-Min Ting, Liyun Chang, Hsiao-Yi Lee, Stephen Wan Leung, Chih-Jen Huang, Pei-Ju Chao

**Affiliations:** 1 Medical Physics and Informatics Laboratory of Electronics Engineering, National Kaohsiung University of Applied Sciences, Kaohsiung 80778, Taiwan, ROC; 2 Graduate Institute of Clinical Medicine, Kaohsiung Medical University, Kaohsiung 807, Taiwan, ROC; 3 Department of Electrical Engineering, National Kaohsiung University of Applied Sciences, Kaohsiung 80778, Taiwan, ROC; 4 Department of Radiation Oncology, Kaohsiung Yuan’s General Hospital, Kaohsiung 80249, Taiwan, ROC; 5 Department of Radiation Oncology, Kaohsiung Chang Gung Memorial Hospital and Chang Gung University College of Medicine, Kaohsiung 83342, Taiwan, ROC; 6 Department of Medical Imaging and Radiological Sciences, I-Shou University, Kaohsiung 82445, Taiwan, ROC; 7 Department of Radiation Oncology, Kaohsiung Medical University Chung-Ho Memorial Hospital, Kaohsiung 807, Taiwan, ROC

## Abstract

We investigated the incidence of moderate to severe patient-reported xerostomia among nasopharyngeal carcinoma (NPC) patients treated with helical tomotherapy (HT) and identified patient- and therapy-related factors associated with acute and chronic xerostomia toxicity. The least absolute shrinkage and selection operator (LASSO) normal tissue complication probability (NTCP) models were developed using quality-of-life questionnaire datasets from 67 patients with NPC. For acute toxicity, the dosimetric factors of the mean doses to the ipsilateral submandibular gland (Dis) and the contralateral submandibular gland (Dcs) were selected as the first two significant predictors. For chronic toxicity, four predictive factors were selected: age, mean dose to the oral cavity (Doc), education, and T stage. The substantial sparing data can be used to avoid xerostomia toxicity. We suggest that the tolerance values corresponded to a 20% incidence of complications (TD_20_) for Dis = 39.0 Gy, Dcs = 38.4 Gy, and Doc = 32.5 Gy, respectively, when mean doses to the parotid glands met the QUANTEC 25 Gy sparing guidelines. To avoid patient-reported xerostomia toxicity, the mean doses to the parotid gland, submandibular gland, and oral cavity have to meet the sparing tolerance, although there is also a need to take inherent patient characteristics into consideration.

Radiotherapy (RT) is commonly used to treat nasopharyngeal carcinoma (NPC) patients. The parotid, submandibular, and minor salivary glands are often incidentally irradiated during the RT course. Xerostomia is a common complication that reduces the patient’s quality of life (QoL) after RT^1^,^2^. Beetz *et al.* found that the mean dose to the parotid and submandibular glands was positively correlated with the toxicities of xerostomia and sticky saliva^3^. The clinical patient data regarding age and baseline xerostomia also showed significance in their study. Therapy-related dosimetric and clinical patient factors showed an association with toxicity in previous studies^3^,^4^,^5^,^6^,^7^.

Advances in the development of RT delivery techniques, recently with helical tomotherapy (HT), have improved the homogeneity and conformality of the dose delivered to the target tumor in NPC patients^8^,^9^,^10^. As a result, there is a significant reduction in the radiation dose to the parotid glands, with better recovery of the parotid and whole mouth saliva flow and improved patient-reported xerostomia compared with conventional RT^11^.

To avoid xerostomia, the Quantitative Analyses of Normal Tissue Effects in the Clinic (QUANTEC) guidelines to limit the probability of severe xerostomia are as follows: at least one parotid gland should receive a mean dose ≤20 Gy, or both parotid glands should receive a mean dose ≤25 Gy^12^,^13^. In our preliminary work, we investigated the significance of radiation dose distributions in the parotid glands in relation to patient-reported xerostomia among NPC patients treated with HT whose mean dose to the parotid glands met the 25 Gy sparing guidelines of QUANTEC. The results revealed that the dose distributions in the parotid glands showed no significant association with the development of patient-reported xerostomia related to salivary dysfunction among patients treated with HT. A sizeable number of NPC patients treated with HT still have xerostomia, although the mean doses to the parotid glands met the QUANTEC 25 Gy sparing guidelines; this raises the issue of identifying other predictive factors that may be of interest for further analysis.

In the present study, we introduced the normal tissue complication probability (NTCP) model to describe the correlations between predictive factors and the probability of side effects. NTCP modeling in RT aims to assign maximal information concerning the correlation between inhomogeneous therapy-related dose distributions and clinical patient factors with the corresponding outcome data in patient populations into few-parametric models^4^,^14^. Xu *et al.* introduced the least absolute shrinkage and selection operator (LASSO) to build NTCP models of xerostomia after three-dimensional conformal radiation therapy (3D-CRT) for head and neck cancer patients^14^,^15^. Our previous study used the LASSO NTCP model to predict the incidence of moderate to severe patient-reported xerostomia among head-and-neck squamous cell carcinoma (HNSCC) and NPC patients treated with fixed-gantry intensity-modulated radiotherapy (IMRT)^7^ and another treatment used for head and neck cancer patients^16^. A disadvantage was the lack of substantial data on submandibular gland sparing and oral cavity sparing; using only dosimetric information from the parotid gland mean dose parameter may have cause decreased sensitivity and specificity. However, the treatment strategies of HT may differ from those of fixed-gantry IMRT. Wiezorek *et al.*^17^ investigated rotational IMRT techniques compared to fixed-gantry IMRT and HT in a multi-institutional planning study for HNC cases. The overall treatment plan quality using HT seemed to be better than other treatment plan system technology combinations. HT techniques resulted in better target dose homogeneity compared with volumetric-modulated arc therapy (VMAT) and step-and-shoot IMRT. For the parotid gland, clear differences in median dose were observed for the different IMRT techniques. Beetz *et al.*^3^,^4^,^5^ reported that NTCP models developed in a population treated with a specific technique cannot be generalized and extrapolated to a population treated with another technique without external validation. Thus, the fixed-gantry IMRT NTCP models cannot be generalized to HT cohorts. Our previous study validated this finding using HNSCC and NPC patient cohorts treated with fixed-gantry IMRT^7^.

The goals of this study were to investigate the incidence of moderate to severe patient-reported xerostomia among NPC patients treated with curative-intent HT whose mean dose to the parotid glands met the 25 Gy sparing guidelines of QUANTEC, and to identify patient clinical and therapy-related dosimetric factors associated with acute and chronic xerostomia toxicity by using LASSO NTCP modeling^7^,^14^,^18^.

## Methods and Materials

### Study population

QoL questionnaire datasets from 67 patients with NPC were analyzed. All of the participants were treated with HT at Kaohsiung Yuan’s General Hospital between September 2010 and May 2014. The QLQ-H&N35 and QLQ-C30 questionnaires were used as the endpoint evaluation. The mean doses to the parotid glands met the QUANTEC 25 Gy guidelines in 54 of 67 patients. The remaining 13 patients were excluded from this study; 9 patients had higher doses to the parotid glands, and 4 patients were suffering from moderate to severe xerostomia at baseline. The characteristics of the patients with NPC are listed in Table 1.

The present study was approved by Kaohsiung Yuan General Hospital’s Institutional Review Board (YUAN-IRB20140923B) and the Kaohsiung Medical University Chung-Ho Memorial Hospital Institutional Review Board (KMUH-IRB-20140134). All participants provided written informed consent, and all experiments were performed in accordance with relevant guidelines and regulations.

### HT techniques

All of the patients were treated with HT as previously described^9^,^19^. All of the patients were immobilized in a tailor-made thermoplastic cast from head to shoulders, and 3-mm-thickness positron emission tomography/computed tomography PET/CT (Siemens Biograph LSO PET/CT; Siemens, Munich, Germany) scan slices of the head and neck were obtained to use for localization of targets and organs at risk (OARs). PET/CT image sets were then transferred to and fused in the MIM software V 6.17 (MIM Software, Inc., Cleveland, Ohio, USA) for contouring. Contours were transferred to HT for IMRT inverse planning. In four of the patients assessed with PET/CT, distant metastases in the bone, mediastinal lymph nodes, and unexpected small neck nodes were detected by a high standardized uptake value (SUV)^19^.

Details concerning the prescribed dose and fractions for the gross tumor volume (GTV), the clinical target volume (CTV) and planning target volume (PTV) were previously described. Briefly, in this analysis, the median (and modal) prescribed dose was 72 Gy to the PTV (PTV_72_), 64.8 Gy to the elective PTV (PTV_64.8_), and 54 Gy to the clinically negative neck region (PTV_54_) with a daily fraction size of 1.8 Gy in terms of three cone-down treatment schemes. Typically for a prescribed dose equal to 72 Gy, the prescription dose was set to (a) 30 fractions containing all three PTVs, (b) six fractions containing PTV_72_ and PTV_64.8_, and (c) four fractions treated with PTV_72_ alone. OARs included seven serial-type organs (serial OARs; brainstem, spinal cord, lenses, eyes, optic nerves, chiasm, and mandible) and three parallel-type organs (parallel OARs; parotids, submandibular glands, and oral cavity). Maximum doses to OARs were optimized individually without compromising the PTV coverage, with at least 95% of the PTV receiving the minimum prescribed dose^9^,^12^. Treatment was delivered in five fractions per week.

With advances in HT, concurrent chemotherapy may be considered according to the patient’s disease status to improve the control rate in NPC. However, the disease itself should not affect the salivary flow or the patient’s perception of salivary flow independent of radiation dose to those salivary glands. To ensure that xerostomia is induced primarily by radiation treatment, patients with moderate to severe xerostomia at baseline should be excluded from analysis^4^.

### Chemotherapy

Concurrent chemotherapy was administered in 37 (68.5%) patients. The regimens used involved a weekly CDDP (cisplatin) or PF (cisplatin + fluorouracil) regimen for two to six courses, or modified regimens according to the patient’s disease status as determined by the medical oncologist^16^.

### QoL evaluation

Chinese versions of the EORTC QLQ-C30 and QLQ-H&N35 questionnaires were used. They were obtained from the Quality of Life Unit, EORTC Data Center, Brussels, Belgium^20^,^21^,^22^. Details concerning the QoL evaluation can be found in previous studies^16^,^20^,^22^. Briefly, a prospective survey of QoL using the European Organization for Research and Treatment of Cancer (EORTC) C30 and H&N35 QoL questionnaires (QLQ-C30 and QLQ-H&N35) was performed on 67 patients with NPC. The patients were asked to complete the questionnaires prior to and during treatment as well as at 1 month and 6 months after IMRT. The EORTC QLQ-H&N35 questionnaire was used to evaluate the analytical endpoint for xerostomia, and the dry mouth item was used for the patient-reported xerostomia analysis. The primary endpoint was defined as moderate to severe xerostomia at 1 month (XER_1m_) and 6 months (XER_6m_) after the completion of RT. Because we were primarily interested in moderate to severe xerostomia induced by RT itself, patients with moderate to severe xerostomia at baseline were checked^3^,^4^,^5^,^16^,^22^.

### Statistical analysis

A multivariable logistic regression NTCP model with LASSO was developed. Details concerning the multivariable logistic regression analysis, with an extended bootstrapping technique, have been described in previous studies^3^,^4^,^5^,^23^. We used 1000 bootstraps for each analysis. Initially, for each patient, 16 candidate predictive factors were included in the variable selection procedure. The candidates included 11 clinical- and 5 therapy-related dosimetric factors. The dosimetric candidate factors were the mean dose (Gy) to the contralateral submandibular gland (Dcs), ipsilateral submandibular gland (Dis) and oral cavity (Doc). The oral cavity includes the surfaces of the inner lips, buccal mucosa, tongue, base of the tongue, floor of the mouth, and palate. Doc represents the radiation effect on the minor salivary glands. We also included the mean doses to the contralateral and ipsilateral parotid glands to validate the association again, although they were previously found to be not significantly correlated with xerostomia; when the mean doses to the parotid glands met the QUANTEC 25 Gy sparing guidelines, five dosimetric dose-volume histogram parameters were included for further analysis. We used the LASSO process to select the optimal number of potential predictive factors for the NTCP predictive model.

NTCP predictive values were calculated for each set of predictive factors based on the multivariable logistic regression coefficients, according to the following formula for each patient^24^:
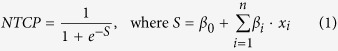
where *n* is the number of predictive factors in the built model; variables *x*_*i*_ represent different predictive factors, and *β*_*i*_ are the corresponding regression coefficients.

The details of the LASSO model can be found in previous studies^14^,^15^,^16^,^25^. The model uses the following equation to shrink the coefficients and select the predictive factors:
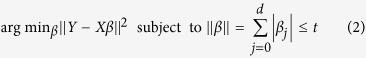
where *d* is the number of variables selected, and *t* is the tuning parameters that control the degree of penalty, which can be determined by cross-validation. Details concerning cross-validation can be found in previous studies^14^,^16^,^26^. However, to generalize the use of the models, a compact model can be generated by manually setting the value of *t* (a penalty setting). In this study, the goal was achieved when the optimal selected number of predictive factors was set to no more than three if the AUC ≥ 0.90. After selecting the predictive factors, the performance measures can be checked using the AUC, scaled Brier score, Nagelkerke R^2^, Omnibus, Hosmer-Lemeshow test and NPV^3^,^4^,^5^,^20^.

Single-mean-dose-NTCP-model-conserved traditional techniques were considered for the most significant dosimetric factors. The parameters for the univariate NTCP regression model are shown for convenience. Statistical analyses were performed using SPSS 19.0 (SPSS, Chicago, IL, USA).

## Results

Fifty-four NPC patients who completed QoL questionnaires at four time points (before RT, during RT, at 1 month and at 6 months after RT) were included in this analysis. The analysis time points were set at 1 month for acute toxicity evaluation and 6 months for chronic toxicity evaluation^27^,^28^.

**Figure 1:**
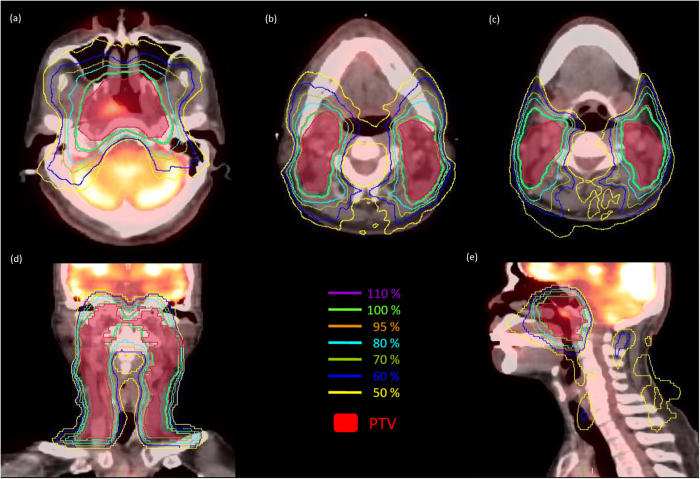
The isodose distributions on transverse, coronal, and sagittal views for one representative nasopharyngeal carcinoma case planned by helical tomotherapy using PET/CT image sets. (**a**) At the nasopharyngeal region. (**b**) At the upper neck region near the parotid glands. (**c**) At the upper neck region near the submandibular glands. (**d**) Coronal view. (**e**) Sagittal view.

**Figure 2:**
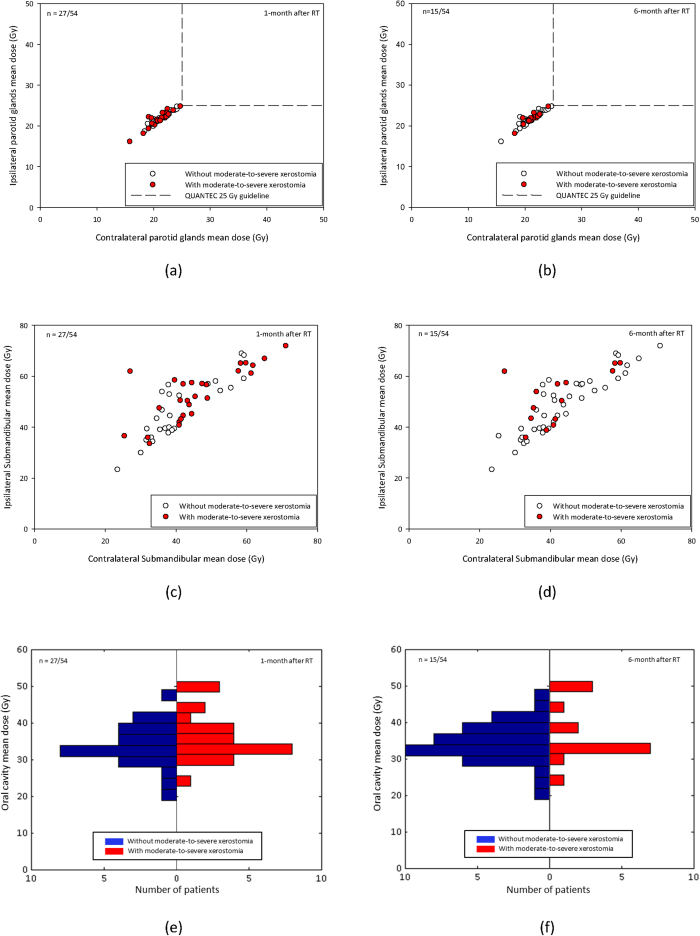
(**a**,**b**) Scatter plots of the ability of the 25 Gy spared contralateral and ipsilateral parotid glands mean dose rule to predict the incidence of xerostomia at the 1-month and 6-month time points, respectively. (**c**,**d**) Scatter plots of the mean dose to the contralateral and ipsilateral submandibular glands at the 1-month and 6-month time points, respectively. (**e**,**f**) Differences in dose distributions of the mean dose to the oral cavity at the 1-month and 6-month time points, respectively. x/x = number of patient with moderate to severe xerostomia/ whole study cohort.

For all of the patients in this study, the HT plans achieved comparable PTV coverage, and the dose-prescription policies were based on the percentage of the prescribed dose that covered >95% of the PTV, and was also equivalent in sparing sensitive structures (parotid glands, submandibular glands, and oral cavity). The isodose distribution of a typical HT NPC patient is shown in Fig. 1. The scatter plots of the mean dose to the parotid glands and submandibular glands for the NPC cohorts are shown in Fig. 2(a–d); additionally, the differences in dose distributions to the oral cavity are shown in Fig. 2(e,f). At 1 month after treatment, 50% (27/54) of the NPC patients reported moderate to severe xerostomia. At 6 months after treatment, 27.8% (15/54) of the patients reported moderate to severe xerostomia, as shown in Table 1. (x /x = number of patient with moderate to severe xerostomia/ whole study cohort).

The initial candidate predictive factors for the patients are shown in Table 2. The variance inflation factor test results for the initial candidate predictive factors were confirmed. There was no multicollinearity between the predictive factors and patients who reported moderate to severe xerostomia.

The LASSO of bootstrap prediction in the multivariable logistic regression analysis ranked the predictive factors in descending order, as shown in Table 3 for both time points. At the 1-month time point, dosimetric factors for the mean dose (Gy) given to the ipsilateral submandibular gland (Dis) and contralateral submandibular gland (Dcs) were selected as the first two significant predictors. The mean dose given to the oral cavity (Doc) followed as the third risk factor. At 6 months, age was the most significant predictor, followed by the mean dose to the oral cavity (Doc), education, and T stage.

The corresponding coefficients of the multivariable logistic regression NTCP models for all of the selected factors are shown in Table 4. The NTCP value for each individual patient can be calculated using logistic regression formulas. For the 1-month time point, four predictive factors were selected, and the NTCP model was S = −48.307 + (Dis * 0.302) + (Dcs * 0.291) + (Doc * 0.566) + (Baseline xerostomia * 1.601); For the 6-month time point, four predictive factors were selected, and the NTCP model was S = −42.149 + (age * 0.548) + (Doc * 0.500) + (Education * corresponding coefficient) + (T stage * corresponding coefficient).

The overall performance for both time points of the NTCP model for patient-reported xerostomia in terms of the scaled Brier score, Omnibus, and Nagelkerke R^2^ was satisfactory and corresponded well with the expected values. The area under the receiver operating characteristic curve (AUC) for the NTCP model discrimination measure was ≥0.95 for both time points. Finally, the calibration measure, namely the Hosmer-Lemeshow test, and calibration slope showed a significant agreement between the predicted risk and observed outcome for both models (Table 5). The negative predictive values are also provided, and the data revealed that there were very low rates of moderate to severe xerostomia.

The parameters for the fitted univariate NTCP regression models, shown in Table 6, were calculated using the most significant predictive factors Dis, Dcs and Doc at the 1-month time point. The acute toxicity tolerance for the ipsilateral and the contralateral submandibular mean dose producing a 50% complication rate (TD_50_) were 50.4 Gy and 43.6 Gy, respectively, for the NPC cohorts 1 month after HT. Additionally, the TD_50_ for Doc was 34.8 Gy. At the 6-month time point, the Doc was the most significant predictive therapy-related factor. The late toxicity tolerance for the oral cavity mean dose producing the TD_50_ was 37.0 Gy for the NPC cohort at 6 months after HT. The tolerances for Dis, Dcs and Doc corresponding to a 20% incidence of complications (TD_20_) are shown in Table 6, when the patients’ mean doses to the parotid glands met the QUANTEC 25 Gy sparing guidelines.

## Discussion

A sizeable number of NPC patients treated with HT still experienced acute xerostomia, even if the mean dose to the parotid glands met the 25 Gy sparing guidelines of QUANTEC (i.e., limiting the probability of severe xerostomia <25% of the pre-RT baseline value fits these criteria): both parotid glands should receive a mean dose ≤25 Gy^13^. In our preliminary study, we investigated the significance of radiation dose distributions in the parotid glands in relation to patient-reported xerostomia among NPC patients treated with HT. After HT, 50.0% of the NPC patients reported moderate to severe xerostomia after 1 month, and 27.8% of the patients reported moderate to severe xerostomia at 6 months. The average mean doses to the ipsilateral and contralateral parotid glands were 21.9 Gy and 21.1 Gy, respectively, for patients without xerostomia; the average mean doses to the ipsilateral and contralateral parotid glands were 21.7 Gy and 21.0 Gy, respectively, for patients with xerostomia. The significance of the relationship between the mean dose to the parotid glands and xerostomia development was low (p > 0.5), because it already met the QUANTEC 25 Gy sparing guidelines. Thus, the major task was to identify other patient and therapy-related factors that may prevent xerostomia toxicity.

The parotid gland mean dose parameter lacks the sensitivity and specificity needed to estimate patient-specific treatment outcomes correctly for NPC patients treated with HT. To increase the predictive performance, additional parameters are required; this study combined clinical patient data and radiation treatment parameters to develop a predictive multicomponent model for xerostomia. In this multivariable model study, the mean doses to the ipsilateral/contralateral submandibular glands and oral cavity were the major components causing xerostomia; however, age, baseline xerostomia, T stage, and education were also selected. These results are similar to the previous study, with the exception of the mean doses to the parotid glands, because the latter had met the criteria to avoid xerostomia provided in the QUANTEC sparing guidelines. This finding is similar to the report by Beetz *et al.*^4^, who also found that the dose to the parotid glands was significantly lower; thus, the relative importance of dose distributions to the submandibular glands increases. Additionally, Eisbruch *et al.* reported that the mean dose to the oral cavity, representing the RT effect on the minor salivary glands, was a significant, independent predictor of xerostomia. Thus, in addition to the major salivary glands, sparing the noninvolved oral cavity should be used as a planning goal to further avoid xerostomia toxicity^18^.

Likewise, we found that elderly patients had a higher probability of developing xerostomia than younger patients who were treated with HT. Older patients are more likely to use medication and have co-morbidities that may influence and reduce saliva production^4^. The patients who had a higher level of education tended to avoid the inconvenience of xerostomia. Similarly, Fang *et al.*^22^ found that NPC survivors with a higher level of education presented a significantly better outcome on QoL scores. The risk was higher with increasing baseline pre-existing minor toxicity^4^,^13^. This multivariate analysis of patient-reported xerostomia clearly indicated that estimation of the risk of developing xerostomia using the NTCP models must be based not only on the dose volume characteristics but also on other potential predictive clinical factors^3^,^4^.

Clinical patient data on normal tissue side effects often include several factors, many of which need to be investigated and considered in a model because they may be related to those side effects. El Naqa *et al.*^23^ showed that prediction of endpoints can be improved by mixing clinical and dose-volume factors, while bootstrap-based variable selection analysis increases the reliability of predictive models. In the present study, the performance of the prediction of patient-reported xerostomia for NPC patients was improved by using multivariable logistic regression models with the LASSO technique. The predictive models selected models with the smallest number of factors while preserving the predictive value with a higher AUC performance. However, the weakness of a multivariate analysis is that the increased complexity may lead to instability of the models, and whether the gain is worth the increased complexity needs further investigation^7^. Other potential weaknesses are as follows: treatment methods may differ among nations and institutions, and different machine settings using the same radiation modality may produce different types and levels of patient-reported xerostomia^3^,^7^.

Chemotherapy was not a significant factor among the candidate predictive factors used in this study, and there was no association between chemotherapy and the risk of patient-reported xerostomia^29^. This finding is similar to the reports of previous studies^12^,^13^,^30^, which showed that the use of chemotherapy was not typically associated with xerostomia toxicity. Our previous findings were also in accordance with these data^7^. However, multiple studies have shown that the use of chemotherapy was typically correlated and increased with the risk of patient-reported xerostomia^31^,^32^. It is difficult to reach any firm conclusion from the literature on the influence of chemotherapy on salivary gland function, because of the lack of standardized registration methods, small sample sizes, relatively short study periods, and different treatment regimens and underlying cancer diagnoses^30^. Chemotherapy regimens may be a risk factor for patient-reported xerostomia, and the correlation with chemotherapy regimens may need to be investigated in the future^33^,^34^.

Schaefer *et al.*^35^ found that Medicare patients with head and neck cancer who were married had better outcomes than similar patients who were unmarried. Marriage was associated with earlier stage, aggressive treatment, and superior survival for patients with oral cavity and pharyngeal cancers^35^. Kissane *et al.*^36^ stated that marriage was as protective as chemotherapy in cancer care. Strikingly, the benefits of marriage were comparable, to or greater than, anticancer treatment with chemotherapy. In the present study, marriage was used as a candidate predictor, but did not show a significant association with patient-reported xerostomia. Regarding education of the study cohort, it was negatively correlated with the toxicities of patient-reported xerostomia. Similar results were reported by Cheng *et al.*^37^, who showed that xerostomia was associated with lower education in a cohort. Because patients with higher education levels are more likely to require additional information regarding their treatment status, uncertainty about xerostomia is relieved. Patients with higher education level showed better recovery at the 6-month time point in this study.

In our previous study, the LASSO NTCP model was used to investigate the incidence of moderate to severe patient-reported xerostomia among HNSCC and NPC patients treated with curative-intent IMRT and to identify clinical and therapy-related dosimetric factors associated with the toxicity^7^. The findings showed that the mean doses to the ipsilateral and contralateral parotid glands were the most important factors in xerostomia; however, age, T stage, financial status and education were also selected. We found that the predictive models developed in the IMRT cohort cannot be generalized and extrapolated to the HT cohort without external validation, and *vice versa*^3^,^7^.

As the mean doses to the ipsilateral/contralateral parotid glands and oral cavity were the three most significant therapy-related dosimetric predictors for the models, single Dis, Dcs, and Doc univariate NTCP regression models were considered. To our knowledge, no univariate NTCP models have been presented for Dis, Dcs, or Doc. For the univariate NTCP analysis, the TD_50_ values for Dis (50% cutoff point) were 50.4 Gy and 52.8 Gy at 1 and 6 months after HT, respectively; the TD_50_ values for Dcs were 43.6 Gy and 49.0 Gy, respectively; and the TD_50_ values for Doc were 34.8 Gy and 37.0 Gy, respectively. The univariate NTCP factors included in the model are useful to further optimize current HT for patient-reported xerostomia and to spare the glands as much as possible.

At the 1-month time point, the incidence of moderate to severe xerostomia complications was 50.0% (n = 27/54); at the 6-month time point, the incidence was 27.8% (n = 15/54), implying recovery^4^,^20^. The recovery effect included the recovery of function of the parotid glands, submandibular glands and other minor glands in the oral cavity. However, the mean doses to the parotid glands are limited by the QUANTEC guidelines, so we provide guidance for setting dose constraints and predicting the risk of moderate to severe xerostomia during treatment planning for the ipsilateral and contralateral submandibular glands. Overall, at the 1-month time point, these data suggest that the tolerance for Dis corresponding to a 20% incidence of complications (TD_20_) was 45.7 Gy, the TD_20_ for Dcs was 38.4 Gy, and the TD_20_ for Doc was 32.5 Gy. For the 6-month time point, the Doc was the only significant dosimetric factor related to toxicity, and the TD_20_ for Doc was 34.5 Gy. A better policy to avoid xerostomia toxicity is to pick the lower tolerance value of those at 1 and 6 months. However, Dis and Dcs were not significant predictive factors, so the tolerance for Dis and Dcs is shown only for reference. These results may indirectly shed light on two important issues raised by Deasy *at al.*^13^: how submandibular sparing should be incorporated into predictive salivary function models and the quantitative effect of oral cavity sparing on xerostomia. These findings imply that the most significant predictive factor for acute xerostomia toxicity is the mean dose to the submandibular glands. Additionally, the factor for chronic toxicity is the mean dose to the oral cavity when NPC patients treated with HT receive a mean dose to the parotid glands ≤25 Gy.

Radiation-induced xerostomia decreases not only QoL but also the compliance of NPC patients in radiation therapy. Careful radiation therapy treatment planning in NPC irradiation can overcome dosimetric issues in PTV coverage and OAR sparing (parotid glands, submandibular glands, and the oral cavity). However, inherent patient characteristics are still involved in the risk for xerostomia toxicity. Thus, more attention should be paid to the elderly, patients with lower education levels, and patients with pre-existing minor complaints at baseline before and after radiation therapy in clinical practice.

## Conclusions

We developed LASSO NTCP predictive models for patient-reported xerostomia in patients treated with HT for NPC cases, where the mean dose to the parotid glands met the 25 Gy sparing guidelines of QUANTEC. We found that both dosimetric information and potential predictive clinical factors need to be considered when estimating the risk of developing xerostomia. Careful radiation therapy treatment planning is needed to simultaneously spare the sensitive OARs and achieve comparable PTV coverage. The predictive factors selected by the LASSO NTCP model are useful to further optimize HT for patient-reported xerostomia and to show which predictive factors are the most important, thus helping spare the glands as much as possible. To avoid patient-reported xerostomia toxicity, not only do the mean doses to the parotid glands have to meet the QUANTEC sparing guidelines, but there is also a need to take the substantial sparing criteria for the submandibular glands and oral cavity into consideration. The available data on submandibular glands and oral cavity sparing can be used to avoid xerostomia toxicity. We suggest a TD_20_ of 39.0 Gy for Dis, a TD_20_ of 38.4 Gy for Dcs, and a TD_20_ of 32.5 Gy for Doc when mean doses to the parotid glands meet the QUANTEC 25 Gy sparing guidelines; picking the lower value of those at 1 and 6 months is the preferable policy to avoid toxicity.

## Additional Information

**How to cite this article**: Lee, T.-F. *et al.* Patient- and therapy-related factors associated with the incidence of xerostomia in nasopharyngeal carcinoma patients receiving parotid-sparing helical tomotherapy. *Sci. Rep.* **5**, 13165; doi: 10.1038/srep13165 (2015).

## Figures and Tables

**Table 1:** Characteristics of patients with NPC treated by HT.

	Value-*n*(%)	
	**Original data (n = 67)**	**Parotid mean dose <25Gy (n = 54)**
Age (y)		
Mean	46	46.4
Range	23–80	23–69
Gender (n)		
Male	55 (82.1%)	45 (83.3%)
Female	12 (17.9%)	9 (16.4%)
T stage		
stage I	28 (41.8%)	22 (40.7%)
stage II	18 (26.9%)	16 (29.6%)
stage III	14 (20.9%)	12 (22.2%)
stage IV	7 (10.4%)	4 (7.5%)
Node classification		
N0	13 (19.4%)	10 (18.5%)
N1+	54 (80.6%)	44 (81.5%)
Mean dose (Range)		
Dip	22.9 Gy (17.2–42.4)	21.8 Gy (16.2–24.9)
Dcp	22.1 Gy (16.8–42.3)	21.1 Gy (15.8–24.7)
Dis	49.4 Gy (23.4–72.0)	49.5 Gy (23.4–72.0)
Dcs	43.2 Gy (23.0–71.0)	43.2 Gy (23.4–71.0)
Doc	34.9 Gy (18.7–51.3)	35.0 Gy (18.7–51.3)
Chemotherapy		
Yes	43 (64.2%)	37 (68.5%)
No	24 (35.8%)	17 (31.5%)
QoL measurement (for XER_1m_)		
With patient-reported xerostomia	31 (46.2%)	27 (50%)
Without patient-reported xerostomia	32 (47.8%)	27 (50%)
With patient-reported xerostomia at baseline	4 (6.0%)	0 (0%)
QoL measurement (for XER_6m_)		
With patient-reported xerostomia	16 (23.9%)	15 (27.8%)
Without patient-reported xerostomia	47 (70.1%))	39 (72.2%))
With patient-reported xerostomia at baseline	4 (6%)	0 (0%)

*Abbreviation:* QoL: quality of life; HT: Helical Tomotherapy; NPC: nasopharyngeal carcinoma.

patient-reported xerostomia was defined as moderate (66) to severe (100) xerostomia 1 and 6 months after the completion of RT, and those patients with moderate to severe xerostomia at baseline were excluded from the analysis.

XER: xerostomia; XER_1m_ or XER_6m_: patient-reported moderate-to-severe xerostomia after 1- or 6-month; Chemotherapy: patient received concurrent chemotherapy, and excluded the patients with patient-reported moderate to severe xerostomia at baseline; Dis: mean dose to the ipsilateral submandibular; Dcs: mean dose to the contralateral submandibular; Dip: mean dose to the ipsilateral parotid glands; Dcp: mean dose to the contralateral parotid glands; Doc: mean dose to the oral cavity.

**Table 2:** Candidate predictive factors initially.

No	Factor	Range or Classification	Median or frequency (XER_1 m_)	Median or frequency (XER_6 m_)	*p-*value (XER_1 m_)	*p*-value (XER_6 m_)
1	Dip	16.2–24.8	21.8	21.8	0.385	0.064
2	Dcp	15.8–24.7	21.1	21.1	0.005	0.068
3	Dis	23.4–72.0	50.5	50.5	<0.001	<0.001
4	Dcp	23.4–71.0	40.9	40.9	<0.001	<0.001
5	Doc	18.7–51.3	33.8	33.8	<0.001	<0.001
6	Age	23–69	46.5	46.5	<0.001	<0.001
7	Marriage	0, 1^#^	15, 39	15, 39	<0.001	0.241
8	Alcohol abuse	0, 1^#^	43, 11	43, 11	<0.001	0.921
9	Smoking	0, 1^#^	28, 26	28, 26	0.002	0.701
10	Chemotherapy	0, 1^#^	17, 37	17, 37	0.008	<0.001
11	Betel nut	0, 1^#^	45, 9	45, 9	0.147	0.734
12	Gender	0, 1^*^	9, 45	9, 45	<0.001	<0.001
13	Baseline xerostomia	0, 1^#^	32, 22	32, 22	<0.001	0.018
14	Node classification	0, 1	10, 44	10, 44	0.001	0.001
15	Education	1, 2, 3	6, 20, 28	6, 20, 28	0.095	<0.001
16	T stage	1, 2, 3, 4	22, 16, 12, 4	22, 16, 12, 4	<0.001	<0.001

*Abbreviation*: Dip: mean dose to the ipsilateral parotid glands; Dcp: mean dose to the contralateral parotid glands; Dis: mean dose to the ipsilateral submandibular; Dcs: mean dose to the contralateral submandibular; Doc: mean dose to the oral cavity; XER_1m_ or XER_6m_: patient-reported with moderate-to-severe xerostomia after 1- or 6month time point.

*0 = Female, 1 = Male; #0 = No, 1 = Yes; Node classification: 0 = N0, 1 = N1, N2, N3; T stage: 1 = T1, 2 = T2, 3 = T3, 4 = T4; Education: E1 = education years <6, E2 = education years 6–12, E3 = education years >12; Baseline xerostomia: 0 = without moderate-to-severe xerostomia, 1 = with moderate-to-severe xerostomia; *p*-value: univariate logistic test.

**Table 3:** Predictive factors correlation ranking for the 1- and 6-month time points by LASSO.

Factor ranking	XER_1m_	XER_6m_
1	Dis	Age
2	Dcs	Doc
3	Doc	Education
4	Baseline xerostomia	T stage
5	T stage	Dis
6	Age	Baseline xerostomia
7	Marriage	Chemotherapy
8	Alcohol abuse	Node classification
9	Gender	Smoking
10	Smoking	Marriage
11	Dcp	Gender
12	Betel nuts	Dcs
13	Chemotherapy	Dip
14	Node classification	Betel nuts
15	Education	Alcohol abuse
16	Dip	Dcp

*Abbreviation:* Dis: mean dose to the ipsilateral submandibular; Dcs: mean dose to the contralateral submandibular; XER: xerostomia; XER_1m_ or XER_6m_: patient-reported moderate- to-severe xerostomia after 1- or 6-month; LASSO: least absolute shrinkage and selection operator; Doc: mean dose to the oral cavity.

**Table 4:** Multivariate logistic regression coefficients and odds ratios for the NTCP for patient-reported moderate- to-severe xerostomia after 1- or 6-month.

	Factors	β	*p*	odd	95%CI
**XER**_1m_	(n = 4)				
	Dis	0.302	<0.001	1.353	1.285–1.424
	Dcs	0.291	<0.001	1.337	1.269–1.409
	Doc	0.566	<0.001	1.762	1.568–1.979
	Baseline xerostomia	1.601	<0.001	4.957	3.121–7.874
	Constant	−48.307	<0.001	0	
**XER**_6m_	(n = 4)				
	Age	0.548	<0.001	1.729	1.587–1.885
	Doc	0.500	<0.001	1.648	1.468–1.851
	Education		<0.001		
	E (1)		<0.001		
	E (2)	−2.189	<0.001	0.093	0.041–0.209
	E (3)	−1.975	<0.001	0.129	0.059–0.283
	T stage		<0.001		
	stage (1)		<0.001		
	stage (2)	−1.165	<0.001	0.312	0.174–0.559
	stage (3)	−1.823	<0.001	0.161	0.081–0.322
	stage (4)	0.160	0.702	1.174	0.516–2.671
	Constant	−42.149		0	

*Abbreviation:* NTCP: normal tissue complication probability; Odd: odds ratio; CI: confidence interval; Dis: mean dose to the ipsilateral submandibular; Dcs: mean dose to the contralateral submandibular; Doc: mean dose to the oral cavity.

XER: xerostomia; XER_1m_ or XER_6m_: patient-reported moderate- to-severe xerostomia after 1- or 6-month.

Education: E (1) = education years <6; E (2) = education years 6–12; E (3) = education years >12; T stage: 1 = T1, 2 = T2, 3 = T3, 4 = T4.

**Table 5:** System performance evaluation.

	XER_1m_	XER_6m_
Brier (scaled)	0.67	0.63
Omnibus	0.001	0.001
R^2^ Nagelkerke	0.77	0.72
AUC (CI95%)	0.96 (0.95–0.97)	0.95 (0.94–0.96)
HL test (*p-value*)	0.73	0.29
Slope-cs	0.97	0.95
Slope-is	0.96	0.97
Slope-oc	0.99	0.91
NPV-*TD*_*50*_	0.89	0.91
NPV-*TD*_*20*_	0.94	0.95

*Abbreviation*: XER_1m_ or XER_6m_: patient-reported moderate- to-severe xerostomia after 1- or 6-month; AUC: Area under the receiver operating characteristic curve; HL: Hosmer–Lemeshow test; NPV: Negative predictive value; TD_50_: the dose predicting a 50% risk of complications; TD_20_: the dose predicting a 20% risk of complications.

Slope-is: The slope of the calibration curve for XER6m-Ipsilateral submandibular gland. Slope-cs: The slope of the calibration curve for XER_6m_-contralateral submandibular gland; Slope-oc: The slope of the calibration curve for XER6m-mean dose to the oral cavity.

**Table 6:** Parameters estimate from the univariate logistic regression NTCP models.

Month	Parameter	TD_50_ (CI95%)	γ (CI95%)	TD_20_(CI95%)
1m	Dis	50.39 (49.06–51.67)	3.75 (2.67–5.04)	45.70 (44.52–46.89)
	Dcs	43.61 (42.18–45.04)	2.94 (2.00–4.05)	38.48 (37.21–39.73)
	Doc	34.83 (34.22–35.49)	5.29 (3.55–7.34)	32.56 (31.98–33.17)
6m	Dis*	52.80 (51.47–54.06)	2.56 (1.44–3.86)	39.02 (37.84–40.21)
	Dcs*	48.99 (47.55–50.41)	3.15 (2.25–4.26)	43.24 (41.98–44.50)
	Doc	37.03 (36.39–37.69)	4.85 (3.10–6.86)	34.56 (33.97–35.17)

*Abbreviation*: NTCP: Normal tissue complication probability; TD_50_: the gland tolerance dose (Gy) that would result in a 50% risk of normal tissue complications for patient-reported moderate- to-severe xerostomia within a specific period of time; γ: the slope of the response curve. Dis: mean dose to the ipsilateral submandibular glands; Dcs: mean dose to the contralateral submandibular glands; Doc: mean dose to the oral cavity; *Factors did not show significant at 6-month time point only show for reference; Grey marks: the lower tolerance value of those at 1 and 6 months.
